# Acute and long-term results with the 3^rd^ generation visually guided laser balloon ablation system for pv isolation

**DOI:** 10.1007/s10840-023-01499-8

**Published:** 2023-02-20

**Authors:** Moritoshi Funasako, Jan Petrů, Pavel Hála, Marek Janotka, Jan Škoda, Milan Chovanec, Lucie Šedivá, Vivek Y. Reddy, Petr Neužil

**Affiliations:** 1 Cardiology Department, Na Homolce Hospital, Roentgenova 2, Prague, 15030 Czech Republic; 2 The Helmsley Electrophysiology Center, Mount Sinai School of Medicine, New York, NY USA

**Keywords:** Laser balloon, Rapid mode, Atrial fibrillation, Pulmonary vein isolation

## Abstract

**Background:**

Visually guided laser balloon ablation is known as an effective pulmonary vein (PV) isolation device. The third-generation laser balloon ablation system (X3) equipped with compliant balloon and an automated motor-driven laser output mechanism, namely RAPID mode, has been clinically proven for PV isolation.

**Methods:**

PV isolation with X3 was performed in all the patients with paroxysmal and early-stage persistent atrial fibrillation (AF). Acute data for PV isolation and clinical outcomes including supraventricular tachyarrhythmia (SVT: AF, atrial flutter, or atrial tachycardia)-free survival rate beyond 1 year were analyzed.

**Results:**

A total of 110 patients (62 ± 13 years old, 80% of paroxysmal AF) were treated with X3. RAPID mode with was utilized to achieve PV isolation in all cases. In combination with RAPID mode and spot mode laser ablation, 91.1% (380/417) of veins were isolated on the first circumferential lesion set and did not require touch-up ablation and during the index procedure 100% of attempted veins were isolated. The mean procedure time was 77.0 ± 22.7 min and LA dwell time was 61.9 ± 22.0 min. Total duration of laser application was 5.1 ± 2.3 min per vein. At 1 year, SVT-free survival rate was 93.7% in paroxysmal AF patients, and 81.1% in persistent AF patients.

**Conclusions:**

A novel continuous automatic laser balloon ablation system was proved to be safe and effective for both paroxysmal and persistent AF patients. The clinical result demonstrated that PV isolation with X3 could achieve a high SVT-free survival rate.

## Introduction

Pulmonary vein (PV) isolation is widely recognized as the gold standard therapy for paroxysmal and persistent atrial fibrillation (AF). Radiofrequency (RF) ablation was first established and played a key role in AF ablation, especially RF PV isolation which has been recognized as an optimal therapeutic option [1–3]. The conventional RF procedure requires experience in catheter handling and output power control, but PV reconnections have been recently reported to occur between 30 and 50% among recurrent AF patients even with highly integrated lesion quality markers/index RF ablation [4–6]. Moreover, overheating of RF applications may create thrombus or steam pops leading to major complications such as ischemic stroke or cardiac tamponade [7]. More recently, balloon ablation technologies have also been developed to simplify the PV isolation procedure and improve clinical outcomes for AF patients [8–11].

**Fig. 1:**
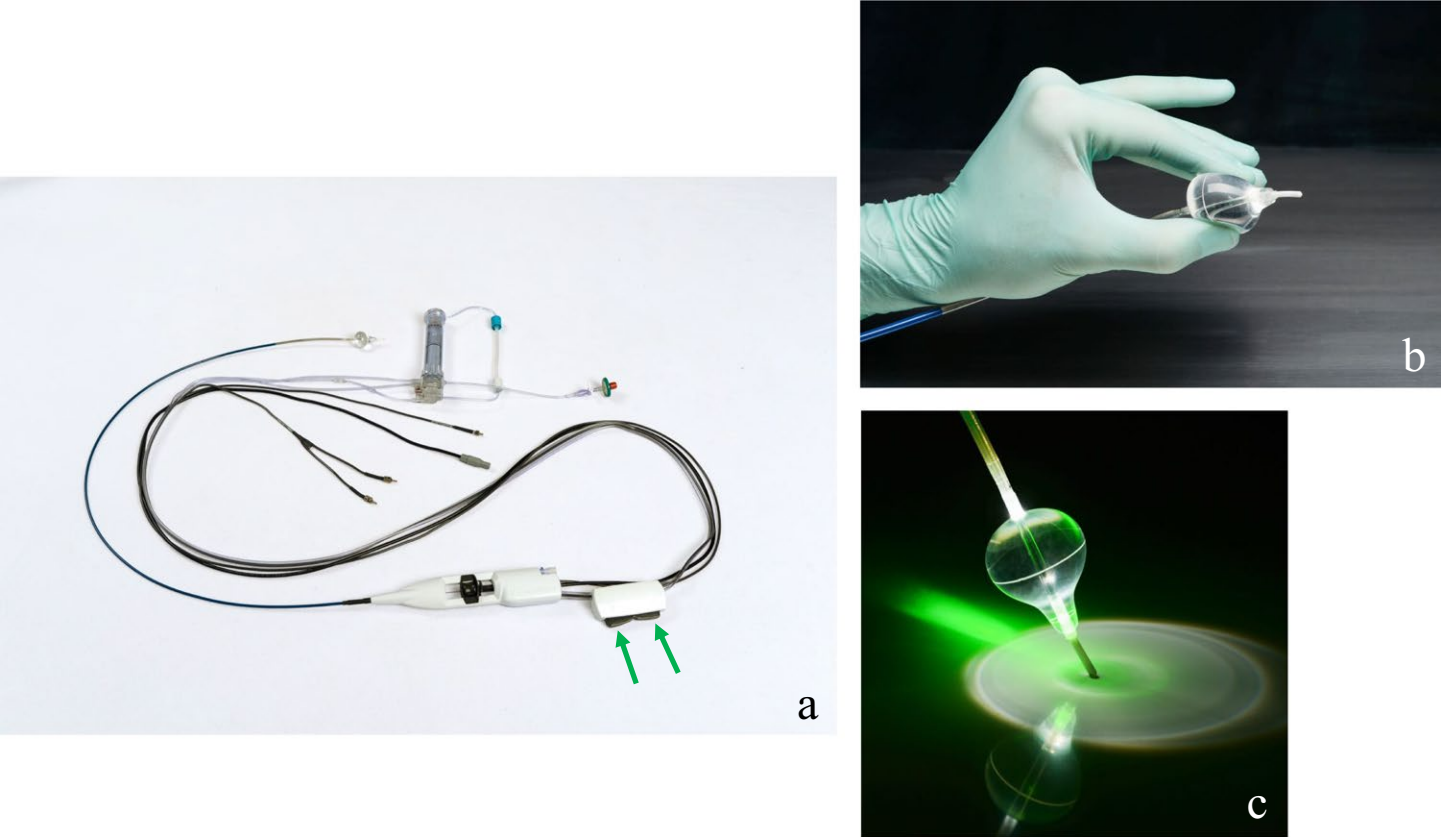
**a** The picture of the X3 laser balloon ablation system. The green arrow shows the controller that enables operators to adjust the balloon size steplessly from 8 to 41 mm. **b**, **c** The pictures of the compliant balloon. Panel **b** demonstrates the compliance of the laser balloon that fits various shapes of PV ostium. Panel **c** shows the laser arc through the compliant balloon aiming at the targeted tissue

Endoscopic visually guided laser ablation for PV isolation (Heartlight®, CardioFocus, Inc., Marlborough, MA, USA) first emerged in 2004 and proved to be a preferable tool to create a transmural lesion in the left atrium with effective and properly titrated laser energy delivery. Laser balloon PV isolation has been known for its safety and durability [12–15]. Combined with a second generation, more compliant balloon compared to the first generation, the latest third-generation laser balloon ablation system (X3) offers visualized tissue contact via an endoscope positioned in the catheter shaft, has proven essential for creating irreversible or durable ablation lesions [16]. Another major feature of the X3 technology is the ability to create continuous lesions with an automated motor-driven, self-rotation laser delivery system, which can create a gapless “single-sweep” lesion around the circumference of the PV in as few as three minutes [17] (Fig. 1).

Our study aims to evaluate the safety and efficacy of PV isolation procedures and clinical results with a novel automatic motor-driven laser balloon X3 ablation system featuring RAPID mode in routine clinical practice.

## Methods

### Patient selection

One hundred and ten (110) consecutive paroxysmal and persistent AF patients were included in the study. The inclusion criteria were defined the same as the standard RF ablation procedure. Exclusion criteria were left atrial (LA) thrombus, left atrium diameter > 60 mm, left ventricular ejection fraction (LVEF) < 35%, previous ablation for AF including PV isolation, NYHA class IV symptoms, recent myocardial infarction within 60 days, unstable angina, uncontrolled bleeding, contraindication of anticoagulation therapy, and active infectious disease. Anatomical characteristics of the LA or targeted pulmonary veins (i.e., left common vein or separated right middle vein/branches) were not considered as an exclusion criterion.

### Ablation procedure

The laser balloon catheter was introduced through the right femoral vein utilizing a dedicated 14 Fr. single deflectable sheath via atrial transseptal puncture. ACT was maintained over 300 s by heparin injection throughout the procedure. The catheter tip was positioned into each vein and confirmed by fluoroscopy and intracardiac echography, then the balloon was inflated by operators with the controller which is located next to the catheter handle. The tissue contact of the balloon was only observed through the endoscopic view. The standard laser output power was set at 13W with RAPID mode where stable contact was obtained without any blood flow on the surface of the balloon, while set at 8.5W, 20 s or 5.5W, 30 s with spot mode ablation was applied where the contact was poor, unstable, or blood flow or blood pool was close to the targeted tissue. Operators were not supposed to deliver laser energy directly on blood. The esophageal temperature was monitored in all the cases and laser energy delivery was terminated when the temperature hit over 41.0°. Phrenic nerve pacing was performed during right PVs isolation to detect phrenic nerve injury as quickly as possible. After all the PV procedures were completed, PV isolation was confirmed by a 20-electrode circular mapping catheter. If PV potentials were still detected after the first attempted encirclement of the PV, the same laser balloon ablation system was used to reisolate the uncompleted vein. All the timeline was recorded from the first groin puncture to the last sheath extraction from the LA.

### Patients’ follow-ups

Oral anticoagulation therapy was continued at least 2 months after the procedure even if the patient was completely free from AF recurrence. The first 3 months after the index procedure was considered as a blanking period when the patient with early recurrence of supraventricular tachyarrhythmia (SVT: i.e., atrial fibrillation, atrial flutter, or atrial tachycardia) was not counted as having a clinically significant recurrence. All the patients with refracting AF who were not able to be treated with an anti-arrhythmic drug or electrical cardioversion were scheduled for a second procedure with a 3D mapping system and standard RF catheter ablation. In the redo procedure, all the PVs were re-evaluated with a 20-electrode circular mapping catheter after the transseptal puncture and insertion of a long sheath. In case of any PV reconnection, RF touch-up ablation of the residual gaps was required to archive PV isolation. Beyond PV isolation strategies (i.e., additional linear ablation and substrate ablation) were considered depending on the operators’ decision.

### Statistical analysis

All the data are expressed as mean ± SD in every table. Differences in frequencies were analyzed by the Chi-square test and a two-sided *P* < 0.05 was considered to be statistically significant (JMP ver. 12.0, SAS Institute Inc.).

### Ethical clearance and data transparency

This study follows the principles of the Declaration of Helsinki. All the patients were informed of and agreed with the purpose of the study and the privacy policy in written form. Ethical approval was acquired by the local institutional ethical committee. The original and additional study data will be made available upon reasonable request.

## Results

### Patient characteristics and PV anatomical features

A total of 110 patients underwent PV isolation with X3. Four-fifths of the patients were paroxysmal (*n* = 88, 80%), the average age was 62 ± 13 years old (paroxysmal: 61 ± 14 years, persistent: 65 ± 11 years), and 37 patients were female (33.6%). More than half of the patients suffered from AF over 1 year (paroxysmal: 64.5%, persistent: 77.8%) and the mean LA size was 43 ± 4.3 mm (42 ± 3.9 mm vs. 45 ± 4.5 mm, paroxysmal vs. persistent AF patients, respectively; *P* = 0.002.). All the patients underwent PV isolation for the first time and 13 patients (12.5%) underwent CT isthmus RF ablation due to prior history of common flutter. The patient characteristics are summarized in Table 1. According to a CT scan or intracardiac echography, the left common vein was confirmed in 20 patients.

**Table 1:** Patient characteristics

Type of AF	Paroxysmal	Persistent	Total
	(*n* = 88)	(*n* = 22)	(*n* = 110)
Age (y.o.)	61 ± 14	65 ± 11	62 ± 13
Male	64.8% (57/88)	72.7% (16/22)	66.4% (*n* = 73)
AF < 1 year	35.5% (27/76)	22.2% (4/18)	33.0% (31/94)
AF > 1 year	64.5% (49/76)	77.8% (14/18)	67.0% (63/94)
Previous AF ablation	0 (0%)	0 (0%)	0 (0%)
Past electrical cardioversion	20.5% (18/88)	40.9% (9/22)	24.5% (27/110)
Hypertension	54.5% (48/88)	40.9% (9/22)	51.8% (57/110)
Coronary artery diseases	5.7% (5/88)	0% (0/22)	4.5% (5/110)
Diabetes	13.6% (12/88)	0% (0/22)	10.9% (12/110)
Past heart failure	2.3% (2/88)	0% (0/22)	1.8% (2/110)
Past cardiac surgery	0% (0/88)	4.5% (1/22)	0.9% (1/110)
Stroke/TIA	3.4% (3/88)	4.5% (1/22)	3.6% (4/110)
Sleep apnea	0% (0/88)	4.5% (1/22)	0.9% (1/110)
AAD before X3 PVI	62.5% (55/88)	94.5% (21/22)	69.1% (76/110)
AAD at the last follow-up	23.9% (21/88)	27.3% (6/22)	24.5% (27/110)

### Procedure details of PV isolation

Of the 420 PVs where treatment was attempted/treated, 417 veins were assessed post-ablation. One patient had four veins, including one untreated vein, and the other three veins were treated but not checked after PV isolation due to interruption of the procedure. The RAPID mode was applied to 418/420 (99.5%) PVs and 138/420 (32.8%) PVs were treated only with the RAPID mode. In combination with RAPID mode and spot mode laser ablation, 91.1% (380/417) of veins were isolated on the first circumferential lesion set. All the veins were isolated only with X3 and no vein required an RF touch-up.

The mean procedure time from the first groin puncture to the sheath extraction from the LA was 77.0 ± 22.7 min. The mean left atrial treatment time (LA dwell time) was 61.9 ± 22.0 min and the mean treatment procedure time was 54.0 ± 20.2 min. In 88.3% of the PVs, RAPID mode was used in most (> 50%) or all of the ablation lesion sets. The mean fluoroscopy time was 3.7 ± 3.2 min. There was no significant difference in procedure time, left atrial treatment time, and fluoroscopy time between paroxysmal AF and persistent AF patients. Total duration of laser application was 5.1 ± 2.3 min (paroxysmal AF: 5.0 ± 2.0 min, vs. persistent AF: 5.8 ± 3.3 min, *p* = 0.03) per vein. In 8 paroxysmal AF patients (7.3%), the catheter was exchanged due to a pinhole during laser application (Table 2).

**Table 2:** Acute results of PV isolation procedure with X3

	Paroxysmal **(***n* = 88)	Persistent (*n* = 22)	Total X3 population (*n* = 110)
Procedure time (min) [1]	76.2 ± 20.7 (88)	80.2 ± 29.5 (22)	77.0 ± 22.7 (110)
Treatment left atrial time (min) [2]	61.8 ± 20.5 (88)	62.1 ± 28.0 (22)	61.9 ± 22.0 (110)
Treatment time (min) [3]	54.4 ± 19.8 (88)	52.7 ± 22.5 (22)	54.0 ± 20.2 (110)
Fluoroscopy time (min)	3.7 ± 3.1 (88)	3.6 ± 3.4 (22)	3.7 ± 3.2 (110)
No. PVs attempted per patient	3.8 ± 0.5 (88)	3.7 ± 0.6 (22)	3.8 ± 0.5 (110)
No. PVs isolated/attempted	100.0% (335/335)	100.0% (82/82)	100.0% (417/417)
No. mapping attempts per PV to achieve isolation			
1	91.6% (307/335)	89.0% (73/82)	91.1% (380/417)
2	7.2% (24/335)	11.0% (9/82)	7.9% (33/417)
≥ 3	1.2% (4/335)	0.0% (0/82)	1.0% (4/417)
No. ablation catheters used			
1	90.9% (80/88)	100.0% (22/22)	92.7% (102/110)
2	9.1% (8/88)	0.0% (0/22)	7.3% (8/110)
Acute isolation rate [4]	100.0% (87/87)	100.0% (22/22)	100.0% (109/109)
First attempt at all veins isolated (FAAVI) [5]	75.9% (66/87)	68.2% (15/22)	74.3% (81/109)
Percent of the vein circumference treated in RAPID			
0%	0.2% (1/419)	0.0% (0/337)	1.2% (1/82)
1–25%	1.4% (6/419)	1.8% (6/337)	0.0% (0/82)
26–50%	9.8% (41/419)	9.2% (31/337)	12.2% (10/82)
51–75%	21.0% (88/419)	20.5% (69/337)	23.2% (19/82)
76–99%	34.6% (145/419)	34.4% (116/337)	35.4% (29/82)
100%	32.9% (138/419)	34.1% (115/337)	28.0% (23/82)
Total therapy time (min) [6]	5.1 ± 2.3 (413)	5.0 ± 2.0 (331)	5.8 ± 3.3 (82)

[1] Defined as the time from venous access to the time of withdrawal of the HeartLight Catheter

[2] Defined as the time from transseptal puncture to withdrawal of the HeartLight Catheter

[3] Defined as the time from the insertion of the HeartLight Catheter to the time of withdrawal of the HeartLight Catheter

[4] Defined as the % of patients where all veins attempted were isolated with the X3 catheter

[5] Defined as the % of patients where all veins attempted were isolated with the X3 catheter and only 1 mapping/vein

[6] Defined as the total number of minutes the laser is active in the vein

The only complications were transient phrenic nerve palsy (PNP) observed in three patients. One of them was the patient without the completed PVI mentioned above, but all PNP recovered at the 6-month visit after the procedures. No other major complications occurred during and after the procedure through 1 year of follow-up.

### Clinical course after PV isolation

**Fig. 2:**
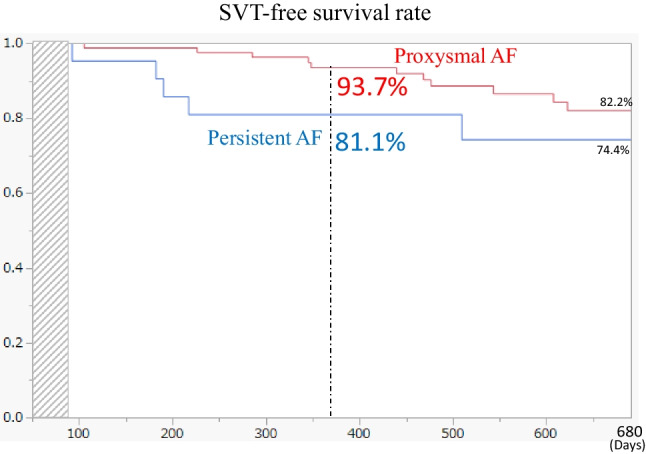
SVT-free survival rate after the index PV isolation with X3. Redline—SVT-free survival rate in patients with paroxysmal AF. Blue line—SVT-free survival rate in patients with persistent AF. One year after the index PV isolation procedure, SVT-free survival rates were 93.7% in patients with paroxysmal AF and 81.1% with persistent AF. Respectively. SVT, supraventricular arrhythmia

All the patients after the first laser balloon PV isolation were investigated employing 12 leads ECG (3 M, 6 M, 12 M), 24 h Holter ECG (6 M, 12 M), and an interview survey on AF symptoms. Recurrent AF is defined as documented sustained AF of more than 30 s by 12 leads ECG or 24 h Holter ECG. Out of 109 patients after PVI, SVT recurrence was observed in 20 patients (14 paroxysmal AF and 6 persistent AF patients) 1 year after the initial procedure and 5 patients underwent redo AF procedures. Of all the 20 recurrent patients, one paroxysmal AF patient underwent electrical cardioversion due to common flutter after the blanking period (109 days after the index procedure) and kept sinus rhythm up to the end of the follow-up period at 15 months. All the other 19 patients developed recurrent AF during follow-up period. At 1 year, SVT-free survival rate was 93.7% in paroxysmal AF patients and 81.1% in persistent AF patients (Fig. 2).

### The findings from the second procedures

**Fig. 3:**
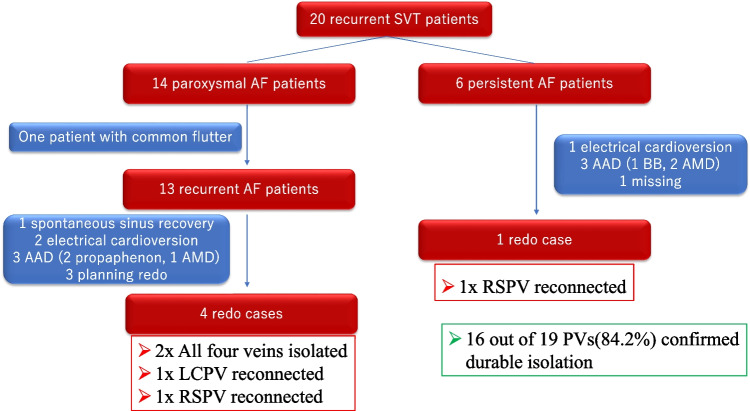
Summary of the performed treatments for recurrent SVTs and the result of electro-anatomical mappings of PVs after the index procedures. Out of 110 patients, 19 AF and one common atrial flutter were observed after the 3-month blanking period. Five patients (4 paroxysmal AF patients and 1 persistent AF patient) underwent the second catheter ablation procedure for recurrent arrhythmias. Among 19 PVs checked with a circular 20-electrode catheter, 16 veins (84.2%) were proved to be isolated. AAD, anti-arrhythmic drag; AMD, amiodarone; BB, beta blockers; LCPV, left common pulmonary vein; RSPV, right superior pulmonary vein

The recurrent AF patients were mainly treated by anti-arrhythmic medications or electrical cardioversion. A total of five patients (4 paroxysmal and 1 persistent AF patients) underwent the second ablation procedure due to the recurrence of AF. Among four paroxysmal patients, two patients had one reconnected PV each (LCPV and RSPV, respectively). One persistent AF patient confirmed reconnected RSPV. Sixteen out of 19 PVs (84.2%) in the recurrent AF patients confirmed durable PV isolation as described in Fig. 3. In the redo cases, additional posterior box isolation, linear ablation, and substrate ablation were performed after PV re-isolation depending on the findings during procedures.

## Discussion

### Rational for durable PV isolation

PV isolation has been recognized as the fundamental procedure for AF treatment. Although “beyond PV” strategies such as linear ablation or substrate-based ablations in addition to PV isolation have been tried, the main factor for clinical AF-free survival results is durable and antral PV isolation [18, 19]. The past studies repeatedly referred to the importance of durable PV isolation as a strong factor for the high AF-free survival rate in any type of AF [20]. Balloon ablation devices have been developed to make PV isolation procedures simple and short; however, durable PV isolation is still challenging as both conventional RF ablation and balloon ablation require experience and technique to control the catheter to get stability and safety profile simultaneously, or to occlude PV with the device in any anatomical conditions, respectively. Different energy sources need specific approaches for PV isolation and laser balloon ablation is known for its safety and durability of PV isolation with a short learning curve [2, 16].

Cryoballoon ablation requires basically total PV occlusion to maximize cryo-thermal effect or a relatively large area with optimal tissue contact as the cryo ablation creates lesions mainly with conductive thermal energy from the balloon surface to the targeted tissue. Laser balloon ablation requires a specifically targeted tissue view via an endoscope as the laser energy through the balloon runs straight directly onto the visualized targeted tissue and generates the heating energy slightly beneath the surface of the tissue with minimal energy loss as long as there is no blood flow or blood pool that may cause intermediate energy attenuation. Laser energy seems to be more concentrated and effective for stable localized transmural ablation lesions wherever the targeted tissue is clearly visible and away from the blood. As an ablation energy source, laser energy has such a unique character that ablation lesions would be minimal but sufficient for durable PV isolation once delivered to the tissue. As seen in this study, this energy-specific lesion formation may have contributed to a high SVT-free survival rate with durable PV isolation and to no left atrial AT recurrences with less reversible ablation lesions that often cause abnormal automaticity, triggered activity, or slow conduction formation leading to clinical ATs.

The latest laser balloon ablation can also reduce the risk of phrenic nerve injury as well when the ablation line is selectively created out of the PV ostium while cryoballoon ablation creates wide lesions all around PV ostium as the northern hemisphere of the balloon generates freezing thermal energy [21]. In this study, three different operators experienced one transient phrenic nerve palsy each during the right superior PV isolation despite phrenic nerve pacing, all three patients recovered to normal function. This implies that the laser energy reached the phrenic nerve to affect the function, but the heating seemed to have been conducive away from the treated tissue where the laser energy was applied. Laser balloon ablation is the only balloon ablation method that enables the design of the isolation line on the actual tissue. Highly selectively designed antral PV isolation lines to avoid the specific location can reduce phrenic nerve injury and esophageal complication in contrast to other single-shot balloon ablation modalities, and at the same time, more antral PV isolation could be archived like RF ablation if necessary.

### Utility of compliant balloon for various anatomical conditions

For balloon ablation modalities, variability in PV anatomy can be a procedural limitation. These balloon techniques require occlusion of the PVs when the energy is delivered to the tissue. Poor tissue contact may lead to unsuccessful PV isolation or reversible lesion formation, and thereby poor clinical results. While cryoballoon ablation is designed to occlude the PV and make wide and circumferential lesions in a single-shot manner with fixed balloon size, laser balloon ablation enables operators to design the antral PV isolation lesion lines via endoscopic view with an expandable compliant balloon adjustable to various PV size from 8 to 41 mm. Specifically, one of the most challenging anatomical profiles is the left common vein [22]. In this study, 20 common veins were all treated only with X3, which is compatible with various PV sizes and shapes. X3 is the only single-sweep device for left common veins compared to other balloon techniques that require multiple segment applications per vein. Right inferior PV is also sometimes challenging for balloon ablations due to poor contact on the bottom aspect. One of the advantages of X3 is the compliant balloon that sits well around the PV ostium and helps create circumferential continuous ablation using RAPID mode even for small veins, common PV trunks, and veins with small or early branches. The laser ablation provides sufficient lesion depth without any pushing maneuver as long as the targeted tissue is visible. Our study showed high flexibility of the X3 system against anatomical anomalies without any RF touch-up ablation.

### Advantage of seamless continuous ablation compared to point-by-point spot mode ablation

The first- and second-generation laser balloon systems did not include the automated rotational RAPID mode ablation feature. Although those prior generations recorded excellent clinical outcomes, each 20- to 30-s single application needs 20 to 50% lesion overlap [13, 14]. The necessity of the repeated overlaps with spot mode ablations between the lesions may make the procedure time long and a higher possibility of a gap or reversible lesion formation would be expected. The newly established RAPID mode provides (1) fast PV isolation as few as three minutes per vein, (2) continuous lesion formation with less possibility to have a gap between two different ablation lesions, and (3) qualitatively stable and durable lesions. Indeed, not only the total procedure time and ablation time but also the first-pass isolation rate was improved compared to the first- or second-generation laser balloon ablation system. As seen in this study, catheter exchanges were required due to a pinhole in 8 cases (7.3%). Since the X3 procedures have been reported as a pivotal study, a relatively high pinhole rate (8–13%) was reported [17, 23]. The RAPID mode may damage the balloon material when applied directly to the blood. Operators should be cautious to choose spot mode ablation if the targeted lesion is next to the blood pool or intermittently covered by blood flow. Despite this limitation, the past study demonstrated that the RAPID mode improved acute PV isolation results with a highly successful PV isolation rate and shorter procedure time compared to the former generation [23]. More recently, the balloon material has been modified to be more robust for RAPID mode usage, which was not clinically available in this study. The new balloon material could improve the percentage of RAPID mode usage, shorten the procedure time, and reduce catheter exchanges, leading to better acute procedural results and long-term patient clinical results.

## Limitations

This study includes some limitations. First, the protocol is a non-randomized, single-arm, and single-center study. Second, the results were not compared with other specific procedures such as RF PVI or cryoballoon PVI. Third, the procedure was limited to PV isolation even for persistent AF patients. Although the number of patients was relatively small, few recurrent arrhythmias were observed beyond 1-year observation. According to the redo cases, in most of the cases, PVs were isolated and non-PV triggers or substrates were considered as the source of AF. Based on this fact, the high durability of the PV isolation with X3 seemed to have contributed to the superb SVT-free clinical result. At the same time, even for persistent AF patients, durable PV isolation should be the essential strategy for better clinical outcomes [24].

## Conclusion

A novel continuous automatic laser balloon ablation system for PV isolation was found to be safe and effective for both paroxysmal and persistent AF patients. The combination of effective laser energy and seamless lesion archives quick and durable PV isolation without serious complication, which was proved in the clinical outcome.

## Abbreviations

AF: Atrial fibrillation
AAD: Anti-arrhythmic drag
AMD: Amiodarone
BB: Beta blocker
LCPV: Left common pulmonary vein
PVI: Pulmonary vein isolation
RF: Radiofrequency
RSPV: Right superior pulmonary vein
SVT: Supraventricular arrhythmia
TIA: Transient ischaemic attack
X3: The third-generation laser balloon ablation system
